# Utility–Leakage Trade-Off for Federated Representation Learning

**DOI:** 10.3390/e27111163

**Published:** 2025-11-15

**Authors:** Yuchen Liu, Onur Günlü, Yuanming Shi, Youlong Wu

**Affiliations:** 1 School of Information Science and Technology, ShanghaiTech University, Shanghai 201210, China; liuych12022@shanghaitech.edu.cn (Y.L.);; 2 Lehrstuhl für Nachrichtentechnik, Technical University Dortmund, 44227 Dortmund, Germany; 3 Information Theory and Security Laboratory (ITSL), Linköping University, 581 83 Linköping, Sweden

**Keywords:** utility, federated learning, differential privacy

## Abstract

Federated representation learning (FRL) is a promising technique for learning shared data representations that capture general features across decentralized clients without sharing raw data. However, there is a risk of sensitive information leakage from learned representations. The conventional differential privacy (DP) mechanism protects the privacy of the whole data by randomizing (adding noise or random response) at the cost of deteriorating learning performance. Inspired by the fact that some data information may be public or non-private and only sensitive information (e.g., race) should be protected, we investigate the information-theoretic protection on specific sensitive information for FRL. To characterize the trade-off between utility and sensitive information leakage, we adopt mutual information-based metrics to measure utility and sensitive information leakage, and propose a method that maximizes the utility performance, while restricting sensitive information leakage less than any positive value ϵ via the local DP mechanism. Simulation demonstrates that our scheme can achieve the best utility–leakage trade-off among baseline schemes, and more importantly can adjust the trade-off between leakage and utility by controlling the noise level in local DP.

## 1. Introduction

The rapid advancement of machine learning has exposed significant practical challenges in traditional approaches, particularly as data volumes generated by edge devices—such as smartphones and IoT sensors—continue to grow exponentially. Conventional machine learning methods, which rely on the centralized aggregation of raw datasets, increasingly struggle with scalability, communication bottlenecks, and operational inefficiency. In response to these limitations, federated learning (FL) [1] has emerged as a pivotal framework addressing inherent weaknesses in traditional machine learning. By avoiding the need to collect raw data in a central location, FL not only mitigates critical privacy concerns and reduces communication overhead but also helps comply with stringent regulatory constraints.

In FL, multiple edge clients jointly train models by computing local model parameters or gradients and sharing them with a central server for aggregation. To improve the generalization capabilities and support various machine learning tasks (like classification or recognition), federated representation learning (FRL) combines the principles of FL and representation learning. It captures the underlying structure of the data and learns robust representations.

Unfortunately, the extracted representations may pose a potential risk of sensitive information leakage. Specifically, if these representations inadvertently encode sensitive attributes—such as demographic or health information—they can become a proxy for reconstructing them, thereby serving as a primary vector for privacy violations. As a fundamental principle of machine learning, privacy seeks to safeguard private and sensitive information—such as patient healthcare records or social network addresses and political affiliations—throughout the entire life cycle of a model, from training to deployment. This work addresses privacy risks arising from model outputs during the deployment phase, which stem from the leakage of sensitive information through the released data. To protect privacy, differential privacy (DP) [2,3,4,5,6,7] is the most popular context-free notion of privacy, and its key idea is to add noise to the output or randomize data to conceal private information before sharing. However, DP would negatively impact model performance and does not provide any guarantee on the average or maximum information leakage [8]. Moreover, in many scenarios, some data information is public or non-private, and only sensitive information (e.g., race and gender) needs to be protected. For example, in AI recommendation applications, some users might share their favorite restaurants to receive tailored recommendations, while others prioritize sensitive information leakage and avoid sharing such information. Information-theoretic (IT) privacy focuses on designing mechanisms and metrics to protect privacy. It uses information theory metrics (like f-divergences and mutual information) to quantify the trade-off between privacy and utility, measuring how much information an adversary can infer about private features from released data. Building upon this theoretical foundation, subsequent research has focused on adapting and applying these principles to privately disclose useful information. As an early exploration, ref. [9] proposed a mutual information-based privacy metric specifically designed for testing the effectiveness of privacy-preserving data mining algorithms. From a different perspective, one seminal work [10] addresses the fundamental problem of disclosing useful information under perfect privacy constraints for a variable *S*. It establishes a necessary and sufficient condition: non-trivial disclosure is possible if and only if the smallest principal inertia component of the joint distribution (*S*, *X*) is zero. Furthermore, the work derives tight bounds for this trade-off and provides explicit constructions of privacy-assuring mappings that achieve these bounds. From a more practical perspective, ref. [11] pursued a data-driven framework for optimizing privacy-preserving data release mechanisms to attain the information-theoretically optimal privacy–utility trade-off. An adversarially trained neural network is introduced to implement randomized mechanisms and to perform a variational approximation of mutual information privacy. Moreover, the privacy–utility trade-off in data release under a rate constraint is investigated in [12], which can be considered as a generalization of both the information bottleneck and privacy funnel problems. A necessary and sufficient condition for the existence of positive utility under perfect privacy is established in this paper. Furthermore, a general family of optimization problems, termed complexity-leakage-utility bottleneck (CLUB), is introduced in [13], which provides a unified theoretical framework that generalizes and unifies a wide spectrum of information-theoretic privacy models.

In this paper, we consider a statistical framework where mutual information serves as the metric for both utility and leakage [10,13,14,15]. Consider a utility–leakage problem with the Markov chain (Y,S)−X−Z, where *X* denotes the random variable of the input data, *Y* represents the target objective (e.g., label), *S* is the sensitive information to be protected, and *Z* denotes the extracted representation *Z*. Information utility and leakage are measured by I(Z;Y) and I(S;Z), respectively. It is worth mentioning that all the aforementioned works focused on classical representation learning, instead of federated representation learning. It is uncertain whether their schemes still preserve sensitive information protection, considering the local model updates, global aggregation, and the complicating factor of data heterogeneity.

In this paper, we propose a leakage-restrained federated learning framework that theoretically guarantees the protection of sensitive information by using ϵ-local DP (LDP) mechanisms on the extracted representation. This can upper bound the maximum information leakage of sensitive information by I(S;Z)≤ϵ, regardless of local model updates, global aggregation, or data heterogeneity. The proof follows by using the data processing inequality and leveraging the connection between ϵ-LDP and mutual information. Furthermore, leveraging the upper bound on maximum information leakage, we propose a simple yet efficient training loss function that involves minimizing the conditional uncertainty H(X|Z,S), offering a simpler alternative to directly minimizing I(S;Z) (intractable for most high-dimensional data sources). Simulation results demonstrate that our scheme can achieve the best utility–leakage trade-off among baseline schemes, and more importantly, can tune the trade-off between leakage and utility by controlling the noise level in local DP.

*Notations*: We denote random variables by capital letters and their realizations by lowercase letters. The probability distribution of a random variable *X* is denoted by $P_{X}$ and its probability density function by $p_{X}\left(x\right)$. Given a finite set S, |S| denotes its cardinality. We may drop the capital letter when it is clear from the context (e.g., $p_{X}\left(x\right)=p\left(x\right)$), and use a subscript to emphasize the dependence of the measures on the choice of distribution parameterization (e.g., $p_{\phi }\left(\hat{z}|x\right)$). The expectation is denoted by E[·]. The Shannon entropy and mutual information are denoted by H(X) and I(X;Y), respectively.

## 2. Problem Formulation

### 2.1. FRL with Sensitive Attribute

**Figure 1:**
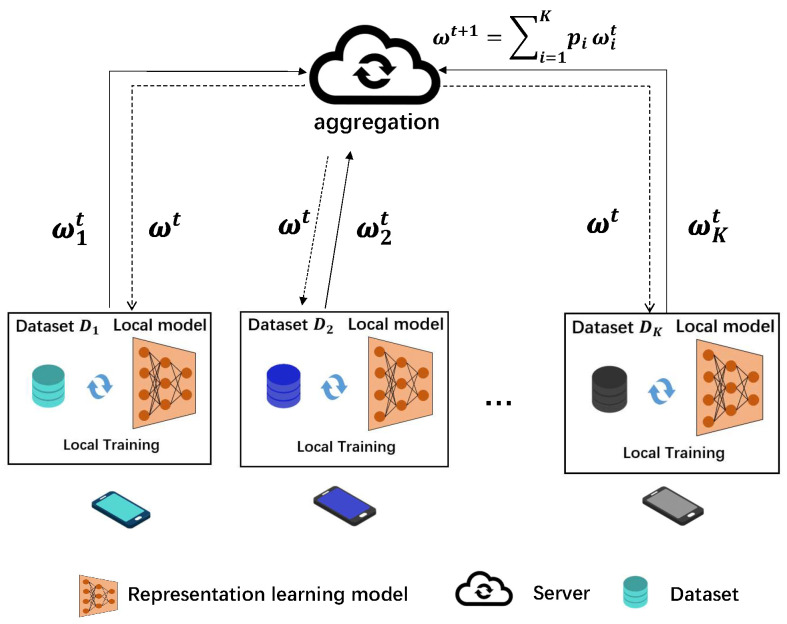
Federated representation learning framework.

Consider an FRL system consisting of one central server and *K* devices indexed by K={1,2,...,K}, as shown in Figure 1.

Each device k∈K has a local training dataset $D_{k}$ with $|D_{k}|$ data samples $\left(x,y,s\right)\in D_{k}$, where *x* is the observed data sample, *y* is the corresponding utility attribute, and *s* is sensitive attribute to be protected (e.g., gender). The entire dataset is denoted by $D=\cup_{k\in K}D_{k}$.

Let l(f(x;ω),s,y) denote the sample-wise loss function on data sample (x,y,s), where f(·,ω) is the model *f* parameterized by ω. The model *f* can be decomposed as f(x;ω)=ϕ(ψ(x))=ϕ(z), where ψ is the encoder, z=ψ(x) is the representation vector, and ϕ is the decoder.

The local loss function of device *k* is given by $L_{k}\left(\omega \right)=\frac{1}{|D_{k}|}\sum_{\left(x,y,s\right)\in D_{k}}l\left(f\left(x;\omega \right),s,y\right).$ Accordingly, the global loss function is given by $L\left(\omega \right)=\sum_{k=1}^{K}\frac{|D_{k}|}{\left|D\right|}L_{k}\left(\omega \right).$ The objective of the federated learning system is to train a global model f(·;ω) that minimizes the global loss L(ω), i.e., $\min_{\omega }L\left(\omega \right)$.

### 2.2. Sensitive Information Leakage–Utility Model

Given input *X*, its representation Z=ψ(X), and its ground-truth label *Y*, we define the utility metric as the mutual information between *Z* and *Y* as follows:

(1) Utility:I(Z;Y)=H(Y)−H(Y|Z).

A higher value of I(Z;Y) indicates that the representation contains more useful information about the target label *Y*. When H(Y|Z)=0, the representation can perfectly estimate *Y*.

Given sensitive information *S* and extracted representation *Z*, we define the sensitive information leakage as

(2) Sensitiveinformationleakage:I(S;Z).

The definition in (2) characterizes the amount of sensitive information contained in the representation relevant to the sensitive attribute *S*. Additionally, by data processing inequality, any operation on *Z* will leak sensitive information no more than I(S;Z).

With the definitions above, we formally define the theoretical guarantee of sensitive information leakage as follows.

**Definition 1.**  *Given any positive value ϵ, an FRL system with sensitive attribute S and representation Z is said to be ϵ-sensitive information leakage if I(S;Z)≤ϵ.*

Our goal is to design an FRL method that maximizes sufficient information for utility, while maintaining the system with sensitive information leakage restriction, i.e., ensuring ϵ-sensitive information leakage guarantee. The optimization problem can be formulated as follows:

(3) $$\begin{array}{cc} \; & \max_{p\left(z|x\right)}I\left(Y;Z\right)\;s.t.\;I\left(S;Z\right)\leq \epsilon . \end{array}$$

**Remark 1.**  *When the sensitive attribute s is associated with fairness attributes such as gender, race, etc., problem (3) can be viewed as a fairness problem where the predictions should be unbiased across different groups. This formulation aligns with the established works [16,17,18,19].*

## 3. Leakage-Restrained Federated Representation Learning

### 3.1. Proposed FRL Framework

Directly solving the optimization problem in (3) is infeasible, mainly because simultaneously maximizing I(Y;Z) and minimizing I(S;Z) via one encoder is challenging, and also because the joint distribution p(s,y,z) is hard to obtain. To address this issue, we alternatively minimize the upper bound on I(S;Z), whose computation does not necessarily explicitly require p(s,z).

Rewrite the mutual information I(S;Z) as follows:

$$\begin{array}{cc} I(X,S;Z) & =I(X;Z)+I(S;Z|X) \end{array}$$

(4) $$\begin{array}{cc} \; & \overset{\left(a\right)}{=}I\left(X;Z\right), \end{array}$$

(5) $$\begin{array}{cc} I(X,S;Z) & =I(S;Z)+I(X;Z|S), \end{array}$$

where (a) holds due to the Markov chain (Y,S)−X−Z. From (4) and (5), we have

(6) $$\begin{array}{c} \begin{array}{cc} I(S;Z) & =I(X;Z)-I(X;Z|S). \end{array} \end{array}$$

By data processing inequality and Markov chain (Y,S)−X−Z, we have I(S;Z)≤I(X;Z). With the observation (6), we aim to upper bound I(X;Z) by ϵ

I(X;Z)≤ϵ,

and this will bring us two important advantages.

- According to Definition 1, I(S;Z)≤I(X;Z)≤ϵ assures that the system is ϵ-sensitive information leakage guarantee.
- If I(X;Z)≤ϵ, then
  (7) $$\begin{array}{c} I\left(S;Z\right)=I\left(X;Z\right)-I\left(X;Z|S\right)\leq \left(\epsilon -I(X;Z|S)\right).\; \end{array}$$
  This enable us to minimize the upper bound of $I\left(S;Z\right)\leq \left(\epsilon -I(X;Z|S)\right)$ as follows:
  (8) $$\begin{array}{cc} \; & \min\left(\epsilon -I(X;Z|S)\right)\\ \Leftrightarrow & \min\left(\epsilon -(H(X|S)-H(X|Z,S)))\right)\\ \overset{\left(a\right)}{\Leftrightarrow } & \min H(X|Z,S), \end{array}$$

where (a) holds since H(Z|S) is a constant given the dataset. Minimizing H(X|Z,S) could be easily solved by constructing a decoder that recovers *X* from representation *Z* and *S*.

To achieve I(X;Z)≤ϵ, we adopt the ϵ-LDP mechanism [20], denoted by $M_{\epsilon }\left(\cdot \right)$, which is defined as follows:

**Definition 2.**  *For any ϵ>0, a randomized mechanism $M_{\epsilon }\left(x\right)$ satisfies ϵ-LDP if and only if for every $x\neq x^{\prime }\in X$ and any measurable set C∈W, it holds*

(9) $$\begin{array}{c} \frac{\mathrm{Pr}[M_{\epsilon }\left(X\right)=C|X=x]}{\mathrm{Pr}[M_{\epsilon }\left(X\right)=C|X=x^{\prime }]}\leq e^{\epsilon }. \end{array}$$

Unlike the conventional privacy FL scheme that adds random noise on the local parameter $\omega_{k}$, we place the ϵ-LDP mechanism after the output of the parameterized feature vector extractor, denoted by $\tilde{\psi }\left(\cdot \right)$. Thus, the process of the model can be presented as a Markov chain

(10) $$\begin{array}{c} \left(Y,S\right)-X\overset{\tilde{\psi }\left(\cdot \right)}{-}V\overset{M_{\epsilon }\left(\cdot \right)}{-}Z, \end{array}$$

where $V=\tilde{\psi }\left(X\right)$ and $Z=\psi \left(X\right)=M_{\epsilon }\left(\tilde{\psi }\left(X\right)\right)$. With the ϵ-LDP mechanism on the extracted feature *V*, we can guarantee the ϵ-sensitive information leakage and achieve I(X;Z)≤I(V;Z)≤ϵ; the detailed proof will be provided later in Section 3.2.

From (7), (8), and the optimization problem in (3), we parameterize the encoding distribution p(z|x) and introduce the following objective function $L(p_{\theta }\left(z|x\right),\beta )$:

(11) $$\begin{array}{c} \begin{array}{c} \min_{p_{\theta }\left(z|x\right)}-I\left(Z;Y\right)\;+\;\beta H\left(X|S,Z\right). \end{array} \end{array}$$

Let $q_{\theta_{1}}\left(y|z\right)$ be a parameterized variational approximation of p(y|z), and $q_{\theta_{2}}\left(x|z,s\right)$ be a parameterized variational approximation of p(x|z,s). The variational upper bound of (11) can be obtained as follows:

(12) $$\begin{array}{cc} -I(Z;Y) & +\beta H(X|S,Z)\\ \; & =-H(Y)+H(Y|Z)+\beta H(X|S,Z)\\ \; & \leq -E_{p_{\theta }\left(x,y,s,z\right)}\left(\log p\left(y|z\right)+\beta \log p\left(x|s,z\right)\right)\\ \; & \overset{\left(a\right)}{=}-E_{p(x,y,s)}p_{\theta }\left(z|x\right)\left(\log p\left(y|z\right)+\beta \log p\left(x|s,z\right)\right)\\ \; & \overset{\left(b\right)}{\leq }-E_{p(x,y,s)}p_{\theta }\left(z|x\right)\left(\log q_{\theta_{1}}\left(y|z\right)\;+\;\beta \log p\left(x|s,z\right)\right)\\ \; & \overset{\left(c\right)}{\leq }-E_{p(x,y,s)}p_{\theta }\left(z|x\right)\left(\log q_{\theta_{1}}\left(y|z\right)+\beta \log q_{\theta_{2}}\left(x|s,z\right)\right), \end{array}$$

where (a) follows from Markov chain (Y,S)−X−Z, (b) holds since $D_{KL}\left(p\left(y|z\right)||q_{\theta_{1}}\left(y|z\right)\right)\geq 0$, and (c) holds since $D_{KL}\left(p\left(x|s,z\right)||q_{\theta_{2}}\left(x|s,z\right)\geq 0\right)$.

Given *N* data points ${\left\{x^{\left(i\right)}\right\}}_{i=1}^{N}$, as well as the corresponding samples of utility and sensitive variables ${\left\{y^{\left(i\right)},s^{\left(i\right)}\right\}}_{i=1}^{N}$, we now form the Monte Carlo estimation for (12) by sampling *M* realizations ${\left\{z^{\left(i,j\right)}\right\}}_{j=1}^{M}$ of representation *z* from $p_{\theta }\left(z|x\right)$ for each data point $x^{\left(i\right)}$. We have the Monte Carlo approximate estimation of (12) as $L(p_{\theta }\left(z|x\right),\beta ,q_{\theta_{1}}\left(y|z\right),q_{\theta_{2}}\left(x|s,z\right))$:

(13) $$\begin{array}{cc} L( & p_{\theta }\left(z|x\right),\beta ,q_{\theta_{1}}\left(y|z\right),q_{\theta_{2}}\left(x|s,z\right))\\ \; & =-\frac{1}{N}\sum_{i=1}^{N}(\frac{1}{M}\sum_{j=1}^{M}[\log q_{\theta_{1}}\left(y^{\left(i\right)}|z^{\left(i,j\right)}\right)\\ \; & \;+\beta \log q_{\theta_{2}}\left(x^{\left(i\right)}|z^{\left(i,j\right)},s^{\left(i\right)}\right)]). \end{array}$$

The loss function $L_{k}$ for client *k* with local dataset $D_{k}$ is

(14) $$\begin{array}{cc} L_{k} & =-\frac{1}{|D_{k}|}\sum_{\left(x,y,s\right)\in D_{k}}\;\;\;\;\;\;\left(\log q_{\theta_{1k}}\left(y|z\right)\;\;\;+\beta \log q_{\theta_{2k}}\left(x|z,s\right)\right), \end{array}$$

where $\theta_{1k}$ and $\theta_{2k}$ are parameters of the utility decoder and side decoder, respectively.

**Figure 2:**
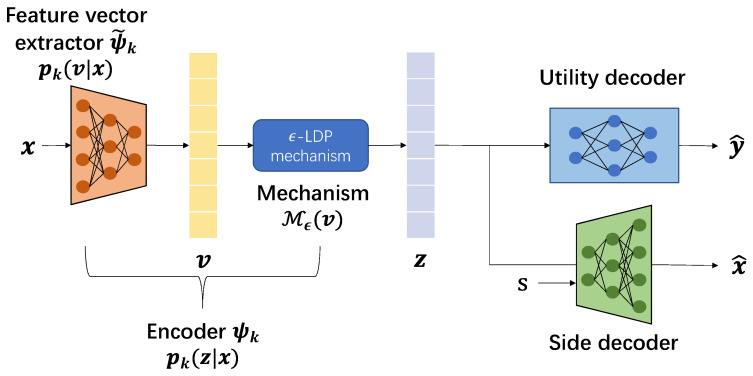
Representation learning model at client *k*.

Based on our proposed loss function (14), we have designed the local learning network at client *k*, as shown in Figure 2. The proposed framework consists of four modules:

- The feature extractor ${\tilde{\psi }}_{k}\left(x\right)=p_{\theta_{k}}\left(v|x\right)$ parameterized by $\theta_{k}$ encodes the original data *x* into feature vector *v*.
- The ϵ-LDP mechanism $M_{\epsilon }\left(\cdot \right)$ maps the feature *v* to an obfuscated representation *z*.
- The utility decoder takes the representation *z* as input and predicts utility variable as $\hat{y}$.
- The side decoder takes both representation *z* and sensitive attribute *s* as inputs to reconstruct input data as $\hat{x}$.

Then, we process the proposed loss function (14) by defining the following:

- $q_{\theta_{1k}}\left(y|z\right)∼B\left(\hat{y}\right)$ (Bernoulli)
- $q_{\theta_{2k}}\left(x|z,s\right)∼N\left(\hat{x},1\right)$ (Gaussian)

The resulting loss function of client *k* in our optimization becomes

(15) $$\begin{array}{cc} L_{k} & =-\frac{1}{|D_{k}|}\sum_{\left(x,y,s\right)\in D_{k}}\;\;\;\;\;\;\left(l_{e}\left(y,\hat{y}\right)+\beta l_{m}\left(x,\hat{x}\right)\right), \end{array}$$

where $l_{e}$ denotes the cross-entropy loss and $l_{m}$ represents the mean squared error (MSE) loss. The complete training framework is outlined in Algorithm 1.
**Algorithm 1** FRL with sensitive information protection.**Input:** Global update rounds *T*, *K* clients, client datasets {$D_{1},D_{2},...,D_{K}$}, learning rate η, initialization model parameters $\omega^{0}=\left\{\theta^{0},\theta_{1}^{0},\theta_{2}^{0}\right\}$**Output:** Aggregated model parameters $\omega^{T}=\left\{\theta^{T},\theta_{1}^{T},\theta_{2}^{T}\right\}$**for** t=0,1,2,...,T−1 **do**    **Server executes:**    Broadcast parameter $\omega^{t}$    Receive ${\left\{\omega_{k}^{t+1}\right\}}_{k=1}^{K}$ from *K* clients    $\omega^{t+1}\leftarrow \frac{1}{K}\sum_{k=1}^{K}\omega_{k}^{t+1}$    **Client *k* executes:**    //*Update local $\omega_{k}^{t+1}=\left\{\theta_{k}^{t+1},\theta_{1k}^{t+1},\theta_{2k}^{t+1}\right\}$*    Receive global model parameters $\omega^{t}$    Update local parameters $\omega_{k}^{t+1}\leftarrow \omega^{t}$    **for** $\left(x,y,s\right)\in D_{k}$ **do**        $v\leftarrow \mathrm{FeatureExtractor}\;p_{\theta_{k}^{t+1}}\left(v|x\right)$        $z\leftarrow \epsilon \text{-}\mathrm{LDP}\;M_{\epsilon }\left(v\right)$        $\hat{x}\leftarrow$**SideDecoder** (z,s;$\theta_{2k}^{t+1}$)        $\hat{y}\leftarrow$**UtilityDecoder** (z; $\theta_{1k}^{t+1}$)        Compute $L_{k}$ from (15), $\omega_{k}^{t+1}\leftarrow \omega_{k}^{t+1}-\eta \nabla L_{k}$    **end for**    return $\omega_{k}^{t+1}$ to server**end for**

### 3.2. Guarantee of Sensitive Information Protection

The following theorem shows that our framework with the ϵ-LDP mechanism on the extracted feature *V* can guarantee the ϵ-sensitive information leakage and achieve I(X;Z)≤ϵ.

**Theorem 1.**  *Consider an FRL framework in Section 3 with sensitive attribute S, feature extractor $\tilde{\psi }:X\to V$, and ϵ-LDP mechanism $M_{\epsilon }\left(\cdot \right):V\to Z$. The representation $Z=\psi \left(X\right)=M_{\epsilon }\left(V\right)$ where $V=M_{\epsilon }\left(\tilde{\psi }\left(X\right)\right)$ satisfies*

(16) $$\begin{array}{cc} \; & I(X;Z)\leq I(Z;V)\leq \epsilon , \end{array}$$

(17) $$\begin{array}{cc} \; & I(S;Z)\leq \epsilon -I(X;Z|S)\leq \epsilon . \end{array}$$

**Proof.**  From the definition of ϵ-LDP, we obtain that $\forall v\neq v^{\prime }$

(18) $$\begin{array}{c} p\left(M_{\epsilon }\left(V\right)=z|V=v^{\prime }\right)\geq p\left(M_{\epsilon }\left(V\right)=z|V=v\right)e^{-\epsilon }. \end{array}$$

Then

(19) $$\begin{array}{cc} p(z)\; & =E_{V∼p(v^{\prime })}\left[p\left(z|v^{\prime }\right)\right]\\ \; & =E_{V∼p(v^{\prime })}\left[p\left(M_{\epsilon }\left(V\right)=z\right)|V=v^{\prime }\right]\\ \; & \geq E_{V∼p(v^{\prime })}\left[p\left(M_{\epsilon }\left(V\right)=z|V=v\right)e^{-\epsilon }\right]\\ \; & =p\left(z|v\right)e^{-\epsilon },\;\;\forall z\in Z,v,v^{\prime }\in V. \end{array}$$

Thus, the mutual information I(Z;V) can be bounded as

(20) $$\begin{array}{cc} I(Z;V)\; & =E_{p(z,v)}\left[\log\frac{p(z,v)}{p(z)p(v)}\right] \end{array}$$

(21) $$\begin{array}{cc} \; & \leq E_{p(z,v)}\left[\log\frac{p(z|v)}{p\left(z|v\right)e^{-\epsilon }}\right]=\epsilon , \end{array}$$

where the inequality follows by (19). With the Markov chain (Y,S)−X−V−Z and (21), by data processing inequality, we have I(X;Z)≤I(V;Z)≤ϵ.

From (6) and (16), we have I(S;Z)≤ϵ−I(X;Z|S). This completes the proof of Theorem 1. □

**Remark 2.**  *The proof requires no assumption on data distributions and model update methods, indicating that Theorem 1 always holds regardless of data heterogeneity. Since the ϵ-LDP mechanism $M_{\epsilon }\left(\cdot \right):V\to Z$ always exists in both the local and global models, the theoretical guarantee I(S;Z)≤ϵ holds for both local and global updates.*

## 4. Simulation Results

In this section, we first present the simulation setting for the FL environment, datasets, simulation metrics, and baselines, and then evaluate the performance of the proposed framework.

Datasets: We perform simulations on two real-world datasets, including an income-prediction dataset, known as the Adult dataset from the UCI Machine learning Repository [21], and the ProPublica COMPAS dataset [22]. The COMPAS dataset comprises 4320 training data samples and 1852 testing data samples, each featuring 11 variables indicating race, age, sex, among others. The Adult dataset comprises 32,561 training data samples and 16,281 testing data samples, each with 14 variables indicating age, workclass, sex, and more.

FL environment: We consider an FRL system with 20 clients and one server. For the COMPAS dataset, each client samples 1500 data samples, and we select race information as the sensitive attribute *S* and recidivism outcome as the utility attribute *Y*. For the Adult dataset, each client samples 6000 data samples, and we choose gender as the sensitive attribute *S* and income as the utility attribute *Y* (predicting whether the annual earning of an individual is more than 50 K per year).

Simulation metrics: Utility performance is measured by mutual information I(Z;Y) and the inference accuracy of *Y*, while leakage performance is measured by mutual information I(Z;S) and the inference accuracy of *S*. I(S;Z) and I(Y;Z) offer direct quantification of the information contained in the representation *Z* about utility label *Y* and the sensitive attribute *S*, which can be regarded as utility information and sensitive information leakage. Consequently, the trade-off between utility and leakage can be quantified by I(Y;Z)−I(S;Z). Mutual information has an intrinsic drawback as a metric: the marginal and joint distributions are typically unknown, making the direct computation of mutual information between two variables intractable. To address this, the Mutual Information Neural Estimator (MINE) maximizes a lower bound on mutual Information as an alternative estimation. However, this approach introduces two key issues:

1. The estimated lower bound may fail to closely approximate the true mutual information, particularly when its actual value is small.
2. Neural network-based estimation can suffer from high variance. This problem is amplified when dealing with high-dimensional data.

To mitigate potential estimation inaccuracies, we employ the inference accuracy of predicting *Y* and *S* from *Z* as another metric. Higher inference accuracy for these variables validates a stronger dependency and indicates that *Z* encapsulates more relevant information about them. In addition, the difference in inference accuracy between *Y* and *S* serves as a complementary metric for evaluating the trade-off.

Baselines: While existing private FL methods defend against privacy attacks during training by addressing privacy risks in gradients and parameters, our approach focuses on reducing the leakage of sensitive attributes from the deployed model’s outputs. Since our method is compatible with most private federated learning approaches, we have selected baseline methods based on representation learning and federated learning to demonstrate its effectiveness. We compare our scheme with the baselines that combine FedAvg [1] with centralized representation learning with sensitive attribute protection, including the privacy funnel optimization-based method PPVAE [23], disentanglement-focused method FFVAE [24], variational approach VFAE [25], and latent distribution learning-based FSNS [26], as well as the raw data without sensitive information protection.

Implementation: We implement the neural network models for the Adult and COMPAS datasets utilizing fully connected layers, where both models share the same architecture presented in Table 1, with input dimension $d_{in}=10$ for COMPAS and $d_{in}=13$ for the Adult dataset. The representation dimensions have been uniformly established as $d_{z}=2$, while the sensitive attribute dimensions have been consistently set to $d_{s}=1$. A one-layer fully connected network is adopted as the inference model for the utility attribute *Y*, while a random forest classifier serves as the inference model for the sensitive attribute *S*, enabling the acquisition of inference accuracy rates for both the utility label *Y* and the sensitive attribute *S*.

**Figure 3:**
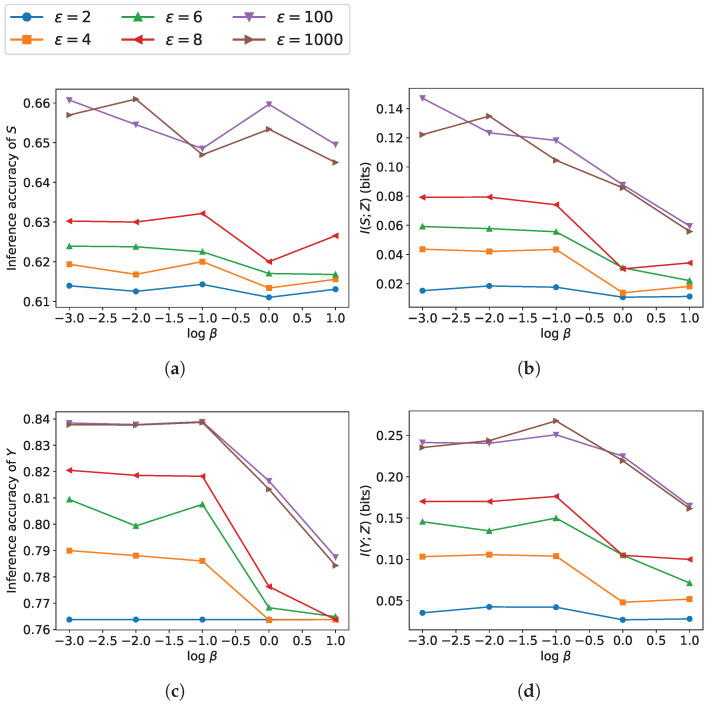
Leakage and utility performance on Adult dataset with $\beta \in \{10^{-3},10^{-2},10^{-1},1,10^{1}\}$: (**a**,**b**) leakage measured via inference accuracy of sensitive attribute *S* (gender) and mutual information I(S;Z); (**c**,**d**) utility measured via inference accuracy of label *Y* (income) and mutual information I(Y;Z).

**Figure 4:**
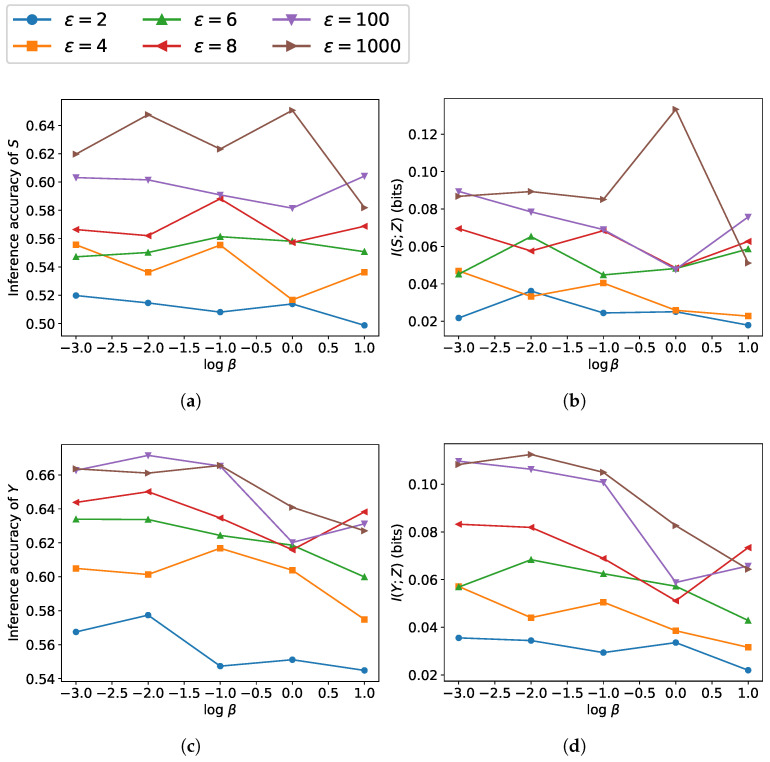
Leakage and utility performance on COMPAS dataset with $\beta \in \{10^{-3},10^{-2},10^{-1},1,10^{1}\}$: (**a**,**b**) leakage measured via inference accuracy of sensitive attribute *S* (race) and mutual information I(S;Z); (**c**,**d**) utility measured via inference accuracy of label *Y* (recidivism outcome) and mutual information I(Y;Z).

Figure 3 and Figure 4 depict the tradeoff between leakage and utility across β as well as ϵ∈{2,4,6,8,100,1000}. Under a higher ϵ, due to the smaller noise scale, β has a greater impact on the model’s performance. As β increases, the performance on sensitive information protection increases while utility performance decreases, which is consistent with the results obtained from our loss function. The reduction in ε leads to a lower upper bound on leakage and a larger noise variance. The increasing noise scale reduces the leakage of sensitive information but degrades the utility. As can be observed, utility performance varies more drastically with changes in β, indicating that compared to leakage, utility is more susceptible to the influence of β.

**Figure 5:**
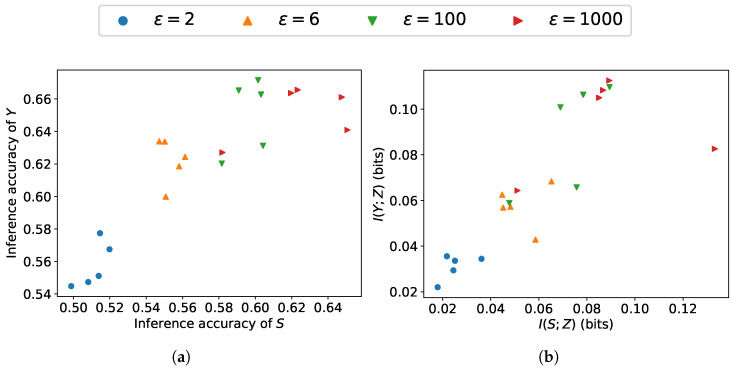
Utility–leakage trade-offs on COMPAS dataset: (**a**) trade-off measured by inference accuracy; (**b**) trade-off measured by mutual information.

**Figure 6:**
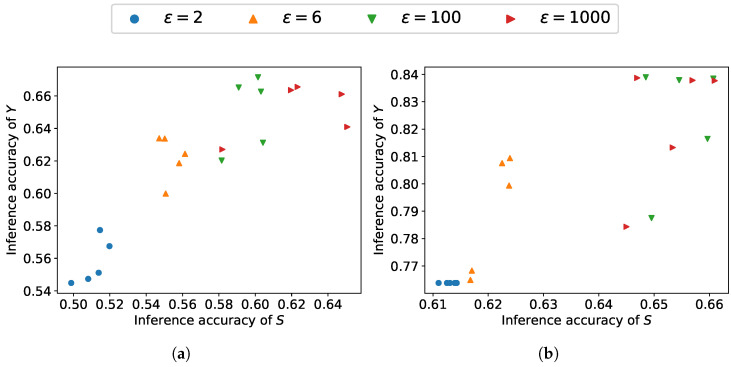
Utility–leakage trade-offs on Adult dataset: (**a**) trade-off measured by inference accuracy; (**b**) trade-off measured by mutual information.

Figure 5 and Figure 6 depict the trade-off between sensitive information leakage and information utility across a range of ϵ when $\beta \in \{10^{-3},10^{-2},10^{-1},1,10^{1}\}$ on the COMPAS and Adult datasets. As the inference accuracy of the utility attribute increases, the inference accuracy of the sensitive attribute also increases, indicating the inherent trade-off between utility and leakage. Given an ϵ, we can attain diverse results by adjusting β. With low ϵ, the proposed scheme can achieve low sensitive information leakage at the expense of utility performance, indicating that less information leakage can be achieved by adding noise at the expense of the utility performance. As can be observed, noise substantially affects the utility–leakage trade-off of the overall model, while the effective range of β is noticeably affected by the magnitude of the noise.

To ensure a fair comparison, we tune the hyperparameters of all compared methods and select the hyperparameters with a similar utility level. Owing to the low dimensionality of the dataset, we chose a dimension of 2 for $d_{z}$ to conduct our experiments. As shown in Table 2, our proposed scheme not only mitigates sensitive information leakage but also achieves a favorable trade-off, as validated by both inference accuracy and mutual information. The failure of FFVAE’s representation learning is evident in label classification accuracy, where classifiers using its extracted representations collapse to predicting only the majority class (all 0 s or 1 s). Consequently, we exclude its performance on other metrics and represent them with “-”.

As can be seen from Table 2, when the representation dimension $d_{z}$ is 2, the utility performance of the compared methods on the COMPAS dataset is already close to that of using raw data directly for inference. However, the disentanglement methods (FFVAE) often require higher dimensions and the compared methods have not fully realized their potential on the Adult dataset, leaving room for enhancement. Therefore, based on the input dimension of two datasets, we conducted further experiments with the representation dimension $d_{z}$ set to 4.

Based on Table 3, it can be observed that FFVAE still performs poorly, while all other methods show improvements in utility. On the COMPAS dataset, the improvement in utility is relatively limited, as the performance of all methods was already strong at $d_{z}=2$. In contrast, the enhancement is more pronounced on the Adult dataset. From the perspective of sensitive information leakage, the inference accuracy of *S* exhibits different trends across the two datasets, with slight increases in some cases and significant decreases in others. On the other hand, mutual information I(S;Z) shows a clear increase in sensitive information leakage, which is reasonable as larger dimensions of feature vectors would potentially incur more information leakage.

We further evaluate the scalability of our method by extending it to accommodate multiple sensitive attributes. This is achieved by generalizing the objective in Equation (11) to include an additional term $H(X|S_{2},Z)$ for a second sensitive attribute, coupled with an additional side decoder dedicated to it. To assess the impact of this extension, we conduct a comparative analysis between configurations with one and two sensitive attributes. We select two distinct sensitive attributes for our evaluation: we designate Prior as the second attribute ($S_{2}$) on the COMPAS dataset, and relationship as $S_{2}$ on the Adult dataset. The first sensitive attribute ($S_{1}$) for each dataset retains the original setting as described in the FL environment. The performance is presented in Table 4.

With the introduction of a second sensitive attribute to protect, our method experiences a decline in both the utility of the data and the level of protection for the first sensitive attribute. This is because we must now strip more sensitive information from the learned representations, and the optimization process must balance preventing leakage from both attributes, which weakens its focus on removing correlations with the original one. Nevertheless, our method successfully strikes a balance, maintaining effective protection against leakage for both sensitive attributes while ensuring an acceptable level of utility preservation.

## 5. Conclusions

In this paper, we focus on FRL that specifically protects sensitive information, such as race or gender. We propose a method that simultaneously maximizes utility information in representations while restricting sensitive information leakage by the LDP mechanism and minimizing the upper bound of sensitive information leakage. We prove that our method theoretically guarantees sensitive information leakage below a predefined positive threshold and empirically demonstrate that it can achieve a better trade-off than baselines.

As further research directions, one could derive a tighter bound for information leakage. A more refined bound for leakage enables the design of more effective schemes for optimizing the balance between data leakage and model utility. In addition, enhancing the robustness of extracted features against noise (perturbation) also represents a highly valuable research direction. Through the augmentation of feature robustness, a simultaneous reduction in information leakage and an improvement in utility performance can be attained when employing noise-based protection mechanisms. Furthermore, the leakage–utility trade-off can be further enhanced by selectively improving the robustness of those features within the feature vector that exhibits lower correlation with the sensitive attribute. Finally, extending the current framework to accommodate scenarios with multiple sensitive attributes constitutes an important and compelling avenue for future research.

## Figures and Tables

**Table 1:** The neural network architecture for Adult or compas datasets.

	Layer	Input	Output
**Encoder**	Dense + ReLU Dense	$d_{in}$ 100	100 $d_{z}$
**Utility Decoder**	Dense + ReLU Dense + Sigmoid	$d_{z}$ 100	100 2
**Side Decoder**	Dense + ReLU Dense	$d_{z}+d_{s}$ 100	100 $d_{in}$
**LDP mechanism**	Laplacian mechanism	$d_{z}$	$d_{z}$

**Table 2:** Utility–leakage trade-off on COMPAS and Adult datasets, $d_{z}=2$.

Dataset	Method	Accuracy (*Y*)	I(Y;Z)	Accuracy (*S*)	I(S;Z)	I(Y;Z)−I(S;Z)	Accuracy (Y)-Accuracy (S)
**COMPAS**	ours	0.6691	**0.1192**	**0.5983**	**0.0098**	**0.1094**	**0.0708**
	FFVAE	0.5377	0.011	-	-	-	-
	PPVAE	0.6632	0.0897	0.9415	0.1485	−0.0587	−0.2783
	VFAE	0.6546	0.0567	0.9834	0.2865	−0.2118	−0.3288
	FSNS	**0.6701**	0.0760	0.6246	0.0207	0.0553	0.0455
	Raw data	0.6776	0.1776	0.6884	0.2506	−0.0729	−0.0108
**Adult**	ours	**0.8389**	**0.1938**	**0.6142**	**0.0325**	**0.1613**	**0.2247**
	FFVAE	0.7637	0.0	-	-	-	-
	PPVAE	0.7879	0.1633	0.7479	0.0769	0.0864	0.040
	VFAE	0.7865	0.1555	0.6672	0.0696	0.0859	0.1193
	FSNS	0.8126	0.2423	0.6689	0.0850	0.1573	0.1437
	Raw data	0.8527	0.3374	0.8391	0.4150	−0.0776	0.0136

**Note:** Bold values denote the best results for each metrics.

**Table 3:** Utility–leakage trade-off on COMPAS and Adult datasets, $d_{z}=4$.

Dataset	Method	Accuracy (*Y*)	I(Y;Z)	Accuracy (*S*)	I(S;Z)	I(Y;Z)−I(S;Z)	Accuracy (Y)-Accuracy (S)
**COMPAS**	ours	0.6717	0.1103	0.6187	**0.0758**	**0.0344**	0.0530
	FFVAE	0.5377	0.0797	-	-	-	-
	PPVAE	0.6627	**0.1384**	0.6639	0.5099	−0.3715	−0.0012
	VFAE	0.6659	0.1154	**0.5910**	0.6574	−0.5420	**0.0749**
	FSNS	**0.6722**	0.1226	0.6293	0.1778	−0.0552	0.0429
	Raw data	0.6776	0.1776	0.6884	0.2506	−0.0729	−0.0108
**Adult**	ours	**0.8364**	0.2136	**0.6595**	**0.0990**	**0.1146**	**0.1769**
	FFVAE	0.7637	0.0	-	-	-	-
	PPVAE	0.8118	0.2262	0.6995	0.2593	-0.0331	0.1123
	VFAE	0.8073	0.2049	0.6684	0.1330	0.0719	0.1389
	FSNS	0.8335	**0.2701**	0.7071	0.2290	0.0411	0.1264
	Raw data	0.8527	0.3374	0.8391	0.4150	−0.0776	0.0136

**Note:** Bold values denote the best results for each metrics.

**Table 4:** Performance comparison of our method with different numbers of sensitive attributes.

Dataset	Method	Accuracy (*Y*)	I(Y;Z)	Accuracy ($S_{1}$)	$I(S_{1};Z)$	Accuracy ($S_{2}$)	$I(S_{2};Z)$
**COMAPS**	ours $S_{1}$ only	0.6691	0.1192	0.5983	0.0098	-	-
	ours $S_{1}$ and $S_{2}$	0.6533	0.0764	0.5890	0.0501	0.6295	0.0178
**Adult**	ours $S_{1}$ only	0.8389	0.1938	0.6142	0.0325	-	-
	ours $S_{1}$ and $S_{2}$	0.8125	0.1485	0.6622	0.0363	0.6986	0.1173

## Data Availability

The data presented in this study are available on request from the corresponding author.
